# Gelatin-Coated TiO_2_/Pd Hybrid: A Potentially Useful Nanomaterial to Enhance Antibacterial and Anticancer Properties

**DOI:** 10.3390/ijms25105308

**Published:** 2024-05-13

**Authors:** Periasamy Anbu, Muruganantham Rethinasabapathy, Anbazhagan Sathiyaseelan, Xin Zhang, Myeong-Hyeon Wang, Sekar Vijayakumar, Yun Suk Huh

**Affiliations:** 1 Department of Biological Engineering, Inha University, Incheon 22212, Republic of Korea; 2 NanoBio High-Tech Materials Research Center, Department of Biological Sciences and Bioengineering, Inha University, 100 Inha-ro, Incheon 22212, Republic of Korea; rmaannauniv@gmail.com; 3 Department of Medical Biotechnology, College of Biomedical Sciences, Kangwon National University, Chuncheon-si 24341, Republic of Korea; sathiyaseelan.bio@gmail.com (A.S.);; 4 Marine College, Shandong University, Weihai 264209, China; vijaysekar05@gmail.com

**Keywords:** titanium oxide nanoparticles, palladium nanoparticles, gelatin, antibacterial activity, anticancer activity

## Abstract

Hybrid nanomaterials have attracted considerable interest in biomedicine because of their fascinating characteristics and wide range of applications in targeted drug delivery, antibacterial activity, and cancer treatment. This study developed a gelatin-coated Titanium oxide/palladium (TiO_2_/Pd) hybrid nanomaterial to enhance the antibacterial and anticancer capabilities. Morphological and structural analyses were conducted to characterize the synthesized hybrid nanomaterial. The surface texture of the hybrid nanomaterials was examined by high-resolution transmission electron microscopy (HR-TEM) and field-emission scanning electron microscopy (FE-SEM). The FE-SEM image revealed the bulk of the spherically shaped particles and the aggregated tiny granules. Energy dispersive X-ray spectroscopy (EDS) revealed Ti, Pd, C, and O. X-ray diffraction (XRD) revealed the gelatin-coated TiO_2_/Pd to be in the anatase form. Fourier transform infrared spectroscopy examined the interactions among the gelatin-coated TiO_2_/Pd nanoparticles. The gelatin-coated TiO_2_/Pd nanomaterials exhibited high antibacterial activity against *Escherichia coli* (22 mm) and *Bacillus subtilis* (17 mm) compared to individual nanoparticles, confirming the synergistic effect. More importantly, the gelatin-coated TiO_2_/Pd hybrid nanomaterial exhibited remarkable cytotoxic effects on A549 lung cancer cells which shows a linear increase with the concentration of the nanomaterial. The hybrid nanomaterials displayed higher toxicity to cancer cells than the nanoparticles alone. Furthermore, the cytotoxic activity against human cancer cells was verified by the generation of reactive oxygen species and nuclear damage. Therefore, gelatin-coated TiO_2_/Pd nanomaterials have potential uses in treating cancer and bacterial infections.

## 1. Introduction

The emergence of antibiotic-resistant bacteria and several types of cancer has become a global health concern, necessitating the development of new antibacterial, anticancer agents or nano-based medicine. Nanomaterials exhibit promising antibacterial and anticancer activities owing to their unique physicochemical properties [1,2]. They also have a high surface-area-to-volume ratio, unique surface chemistry, and increased reactivity, which can enhance their interactions with bacterial or cancer cells, leading to increased antibacterial and anticancer potential. Various nanomaterials, including copper, silver, titanium dioxide, and zinc oxide nanoparticles, displayed antibacterial activity against Gram-positive and Gram-negative bacteria [3,4,5,6] and anticancer potential against different types of cancer cells [7,8]. The nanomaterials can exert their antibacterial effects through several mechanisms, including disrupting the bacterial cell membranes, inhibiting bacterial respiration, and induction of oxidative stress, leading to bacterial cell death. Furthermore, they can inhibit bacterial biofilm formation, which is a significant factor in bacterial resistance to antibiotics.

The unique properties of titanium oxide (TiO_2_) nanoparticles, such as their high surface area, photocatalytic activity, and UV absorption, have attracted considerable attention in several fields, including material science, nanotechnology, and environmental science. Moreover, TiO_2_ nanoparticles are used in medicine and healthcare for drug delivery, medical imaging, and cancer treatment because of their biocompatibility and capacity to transport and release medications at specific locations in the body [9,10]. Palladium nanoparticles (Pd) have attracted attention in industry and research owing to their versatility and catalytic efficiency. High surface area and reactivity make them useful in many applications ranging from material science and healthcare to energy and environmental-related technologies [11,12]. In addition, Pd nanoparticles have potential biomedical applications, including drug delivery systems and photothermal therapy for cancer treatment [13]. Nanoparticles can be used in medical and pharmacological settings because of their high biocompatibility. Although Pd and TiO_2_ nanoparticles can exhibit antibacterial and anticancer properties, increasing the production of reactive oxygen species by hybrid nanomaterial can effectively eliminate harmful bacteria and cancer cells.

Polymer-conjugated nanomaterials have attracted considerable attention for their antibacterial and anticancer effects owing to their unique properties and potential for specialized therapeutic uses [14,15,16]. Biopolymeric materials are eco-friendly, non-toxic, biocompatible, biodegradable, affordable, and easily accessible from natural resources [17]. In particular, gelatin, a protein derived from collagen, is used to spread and stabilize nanoparticles in colloidal suspensions and as a surface modifier to stop the agglomeration of metal nanoparticles. Gelatin-conjugated nanomaterials might enhance the antibacterial and anticancer properties of pharmaceuticals [18,19]. In addition, gelatin-coated nanomaterials play a crucial role in enhancing the performance of white light-emitting diodes [20], developing the nanotherapeutic delivery system for doxorubicin-based cancer treatment [21], and stimulating bone repair [22]. This study examined the antibacterial and anticancer activities of gelatin-coated TiO_2_/Pd hybrid nanomaterials. The synthesized gelatin-coated TiO_2_/Pd was characterized using field-emission scanning electron microscopy (FE-SEM), high-resolution transmission electron microscopy (HR-TEM), Fourier-transform infrared (FT-IR) spectroscopy, X-ray diffraction (XRD), Energy dispersive X-ray spectroscopy (EDS), and X-ray photoelectron spectroscopy (XPS). The gelatin-coated hybrid nanomaterials were tested for anticancer activity against A549 lung cancer cells and antibacterial activity against *Escherichia coli* and *Bacillus subtilis* and were compared with those of the individual constituent materials of the composites.

## 2. Results and Discussion

### 2.1. Physical Characterization

**Figure 1:**
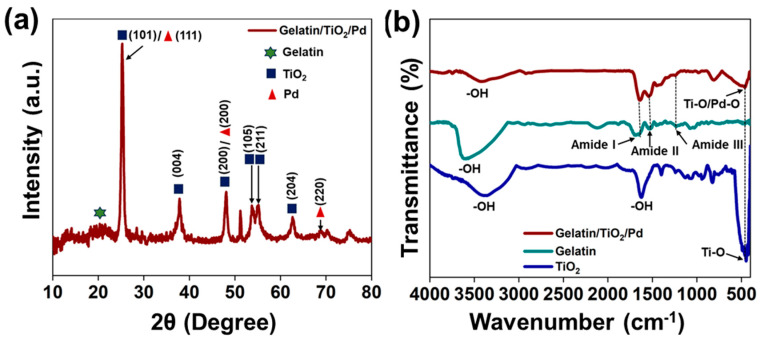
(**a**) XRD patterns of gelatin-coated TiO_2_/Pd hybrid nanomaterials and (**b**) FT-IR spectra of gelatin, TiO_2_, and gelatin-coated TiO_2_/Pd.

In the XRD pattern of gelatin-coated TiO_2_/Pd nanomaterials (Figure 1a), the XRD peak at 20.5° 2θ was attributed to gelatin [23]. The XRD pattern of gelatin-coated TiO_2_/Pd nanomaterials revealed XRD peaks at 25.22°, 37.85°, 48.16°, 53.63°, 55.13°, and 62.72° 2θ, which were indexed to the (101), (004), (200), (105), (211), and (204) planes, respectively, of well-crystallized TiO_2_ (JCPDS card No. 21-1272) in anatase, which is in accordance with previous results [23,24,25,26,27]. Incorporating nanoparticles might destroy the hydrogen bonds between gelatin molecules, allowing TiO_2_ nanoparticles to insert and disperse uniformly in the nanocomposite matrix, strengthening the interaction between nanoparticles and protein molecules [23]. The average crystallite size of TiO_2_ nanoparticles was determined using the Debye–Scherrer formula (Equation (1)) as follows:

(1) $$D=\frac{K\lambda }{\beta cos\theta }$$

where *D* is the crystallite size; λ is the wavelength of the X-ray radiation; K is the Scherrer constant; β is the full width at half maximum (FWHM); and θ is the Bragg’s diffraction angle [28]. The mean crystallite size determined using the Debye–Scherrer equation from the full width at half maximum (FWHM) of the distinct peak at 25.22° 2θ was 13.72 nm, whereas the XRD pattern of the gelatin-coated TiO_2_/Pd composite shows peaks at 25.22°, 37.85°, and 69.2°, which may be attributed to the (111), (200), and (220) planes of Pd [24]. The XRD pattern of the gelatin-coated TiO_2_/Pd composite indicated the successful coverage of gelatin with TiO_2_ and Pd nanoparticles.

The FTIR spectra of gelatin and TiO_2_ were investigated to explain the interactions between the gelatin, TiO_2_, and Pd particles. The gelatin molecule has a wide adsorption band at 3610 cm^−1^ because of the –OH and –NH stretching vibrations [29,30]. Furthermore, the characteristic peaks at 1651, 1529, and 1238 cm^−1^ correspond to amides I, II, and III, respectively (Figure 1b). The amide I band is mainly due to C=O stretching/hydrogen bonding coupled with O–C=O. The amide II and III bands were caused by the bending vibration of –NH and the stretching vibration of –C–N, respectively [23,29,31]. The FTIR spectrum of TiO_2_ nanoparticles showed peaks at 461 and 1612 cm^−1^, which may be assigned to the bending vibration of O–Ti–O in the TiO_2_ lattice and bending vibrations of the –OH group, respectively [24,25,26,27]. Compared to the FTIR spectrum of gelatin, gelatin-coated TiO_2_/Pd shows additional stretching vibration corresponding to metal oxides (Ti–O/Pd-O) at 464 cm^−1^ in addition to the amide-I, -II, and -III peaks of gelatin, confirming the successful formation of gelatin/TiO_2_/Pd. The FTIR spectrum of palladium acetate is provided in the Supporting Information (Figure S1).

**Figure 2:**
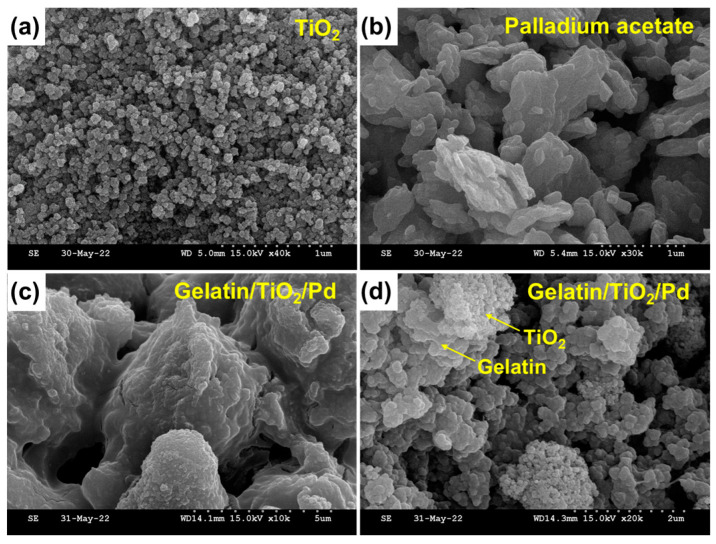
FE-SEM images of (**a**) TiO_2_ nanoparticles, (**b**) palladium acetate, and (**c**,**d**) gelatin-coated TiO_2_/Pd.

The surface textures of TiO_2_, Pd precursor (palladium acetate), and gelatin-coated TiO_2_/Pd were studied by FE-SEM and HR-TEM. Figure 2a shows the FE-SEM image of TiO_2_ nanoparticles, which were agglomerated as small spherical granules. Figure 2b shows an FE-SEM image of palladium acetate. The FE-SEM image (Figure 2c,d) of the gelatin-coated TiO_2_/Pd nanomaterials revealed the presence of TiO_2_ and Pd on the gelatin surface. The EDS mapping (Figure S2) and spectral analysis of gelatin-coated TiO_2_/Pd composite revealed the presence of Ti, Pd, C, and O. The elemental composition (atomic and weight percentages) was also tabulated (Figure S2, inset) in the respective EDS spectra.

**Figure 3:**
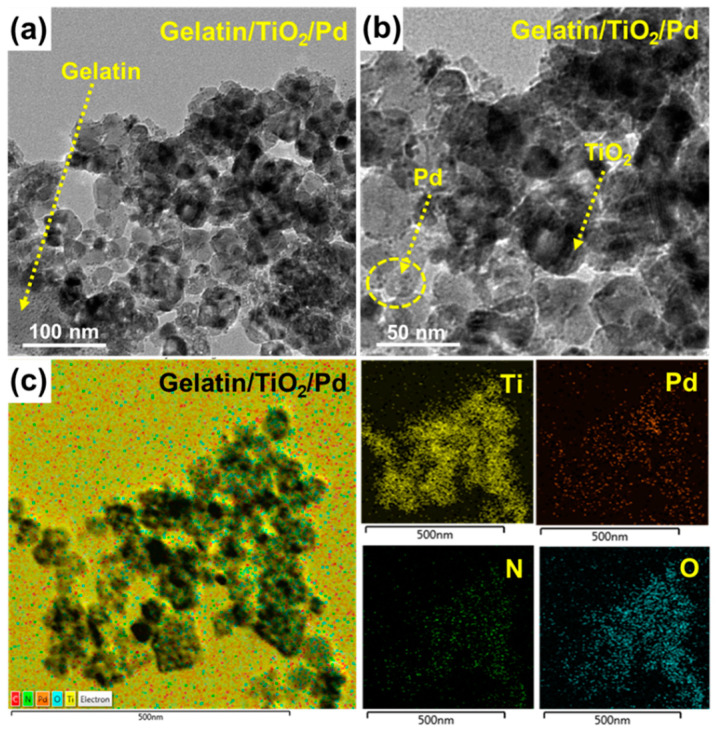
HR-TEM images of (**a**,**b**) gelatin-coated TiO_2_/Pd and (**c**) electron mapping images of gelatin-coated TiO_2_/Pd containing titanium, palladium, nitrogen, and oxygen.

Figure 3a,b present HR-TEM images of gelatin-coated TiO_2_/Pd, showing a layer-structured polymeric morphology of gelatin onto which the TiO_2_ and Pd nanoparticles are anchored homogeneously. HR-TEM images of gelatin/TiO_2_/Pd (Figure 3a,b) revealed mono-dispersed TiO_2_ nanoparticles with almost spherical-like morphologies whose particle sizes ranged between 12 and 23 nm with a mean size of 17 nm, which is in good agreement with the XRD pattern. The TEM images of gelatin-coated TiO_2_/Pd (Figure 3a,b) revealed the uniform distribution of Pd nanoparticles with particle sizes between 2 and 4 nm. The EDS elemental mapping image (Figure 3c) and EDS spectrum (Figure S3) of the gelatin-coated TiO_2_/Pd revealed Ti, Pd, N, C, and O.

**Figure 4:**
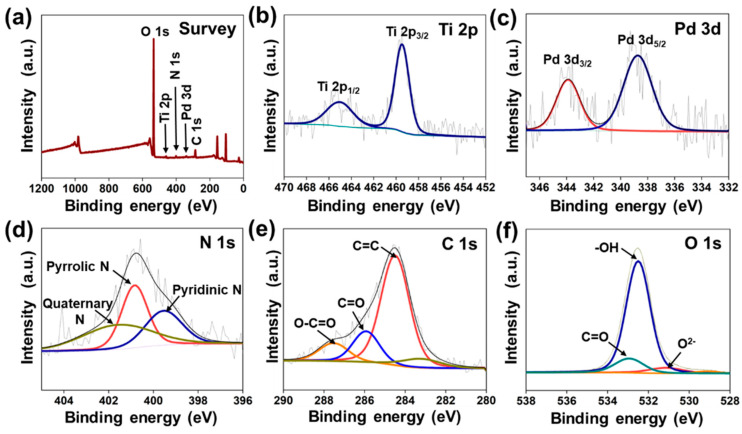
XPS spectra of (**a**) full scan survey spectra, (**b**) Ti 2p, (**c**) Pd 3d, (**d**) N 1s, (**e**) C 1s, and (**f**) O 1s of gelatin-coated TiO_2_/Pd.

XPS was used to confirm the chemical compositions and electronic states of elements. The survey spectrum of gelatin-coated TiO_2_/Pd (Figure 4a) revealed titanium (Ti 2p), palladium (Pd 3d), nitrogen (N 1s), carbon (C 1s), and oxygen (O 1s). Because of the atomic spin-orbit interactions, the high-resolution Ti 2p spectrum was split into two peaks at 459.51 and 465.08 eV (Figure 4b), corresponding to Ti 2p_3/2_ and Ti 2p_1/2_, respectively. The high-resolution peak fitting spectrum of Pd 3p revealed two peaks at 338.73 and 343.89 eV, attributed to the Pd 3d_5/2_ and Pd 3d_3/2_ spin-orbit peaks of Pd (0), respectively, as shown in Figure 4c. Therefore, Pd nanomaterials are present in the zero (0) oxidation state [32]. The XPS N 1s spectra were deconvoluted into three peaks centered at 399.48, 400.82, and 401.71 eV (Figure 4d), which were assigned to pyridinic nitrogen, pyrrolic nitrogen, and quaternary (graphitic) nitrogen, respectively [33]. The C 1s spectrum of gelatin-coated TiO_2_/Pd was deconvoluted into three components centered at 284.52, 285.93, and 287.57 eV, corresponding to C=C, C=O, and O–C=O bonds, respectively (Figure 4e). The O 1s spectrum of gelatin/TiO_2_/Pd was deconvoluted into three components due to the presence of multiple oxygen-containing species. The peaks at 530.67, 531.49, and 53.62 eV were assigned to lattice oxygen, –OH, and –C=O bonds (Figure 4f). In addition, the zeta potential negative value (−21.64 mV) confirmed the significant stability of the hybrid nanoparticles (Figure S4).

### 2.2. Antibacterial Potential

**Figure 5:**
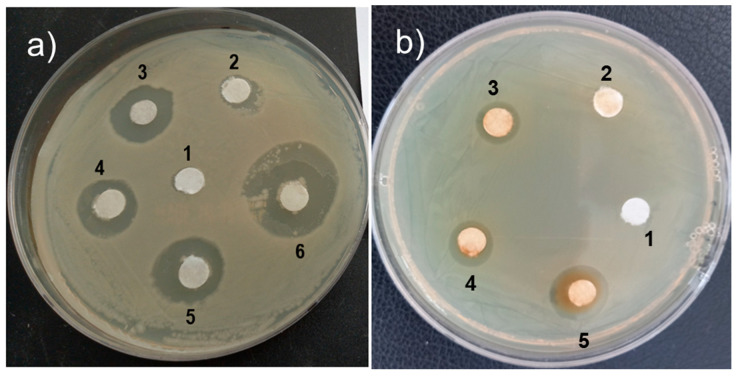
Antibacterial activity of the gelatin-coated TiO_2_/Pd against (**a**) *E. coli* (1, control; 2, TiO_2_; 3, Pd; 4, 15; 5, 30; 6, 45 µg/mL of gelatin-coated TiO_2_/Pd) and (**b**) *B. subtilis* (1, TiO_2_; 2, Pd; 3, 15; 4, 30; 5, 45 µg/mL of gelatin-coated TiO_2_/Pd).

**Figure 6:**
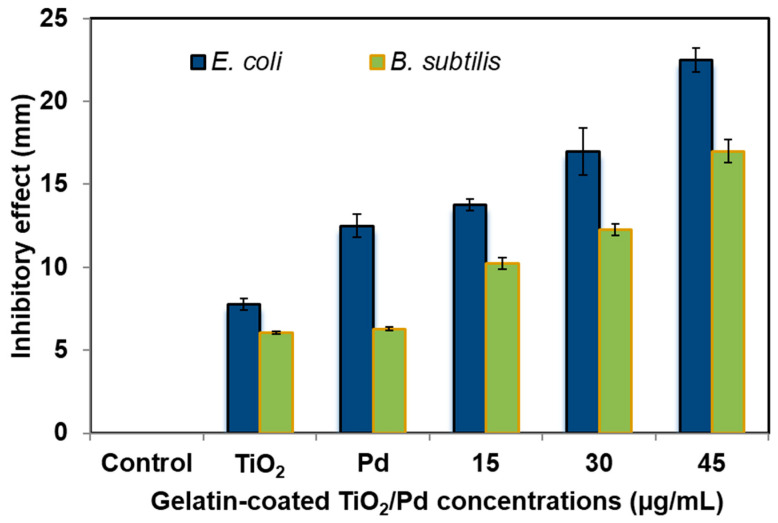
Bar chart showing the inhibitory activity against *E. coli* and *B. subtilis*.

The antibacterial efficacy of the TiO_2_, Pd, and gelatin-coated TiO_2_/Pd hybrid nanomaterial was investigated using disc diffusion studies with *B. subtilis* and *E. coli.* The antibacterial potential of the nanomaterial was demonstrated by its capacity to produce a sizable zone of inhibition surrounding the disc immersed in the material on the plate containing the bacteria. The most potent nanomaterial concentration (45 µg/mL) showed an inhibition zone of approximately 22 mm against *E. coli,* suggesting that the gelatin-coated TiO_2_/Pd is particularly effective at stopping bacterial development (Figure 5a and Figure 6). The significant inhibitory zone was also identified at other concentrations, including 15 and 30 µg/mL, as 14 and 17 mm, respectively. Therefore, the hybrid nanomaterials, even at low concentrations, were more effective at suppressing *E. coli* than TiO_2_ (7.5 mm) and Pd (12.5 mm) alone.

The inhibitory activity of the *B. subtilis* was also checked with different concentrations of hybrid nanomaterials. At a high dosage of 45 µg/mL, *B*. *subtilis* developed inhibitory zones of 17 mm (Figure 5b and Figure 6). At 15 and 30 µg/mL, the low inhibitory activity was 10 and 12 mm, respectively. On the other hand, TiO_2_ and Pd had very low inhibitory zones of *B. subtills*. This result confirmed that the gelatin-coated TiO_2_/Pd hybrid nanomaterial affects *E. coli* growth more than *B. subtilis*. The hybrid nanomaterials can enter the *E. coli* cell easily and inhibit its growth and function because it has a thin peptidoglycan layer on its cell wall [34]. Moreover, TiO_2_/Pd nanomaterials can produce reactive oxygen species inside the bacterial cells, oxidatively damaging macromolecules and causing cell damage and death [35].

### 2.3. In Vitro Cell Viability Assay

**Figure 7:**
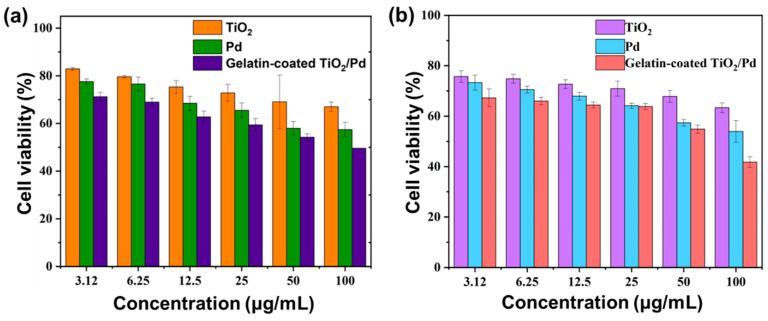
(**a**) Cell viability of NIH3T3 cells at various concentrations of TiO_2_, Pd, and gelatin-coated TiO_2_/Pd. (**b**) Cell viability of A549 cells at various concentrations of TiO_2_, Pd, and gelatin-coated TiO_2_/Pd.

The cytotoxic effects of the gelatin-coated TiO_2_/Pd were assessed using typical NIH3T3 mouse embryonic cells. DMEM was used as the control medium for the cell culture. Figure 7a shows the cell viability of NIH3T3 cells treated with gelatin-coated TiO_2_/Pd. The cell viability was evaluated at different concentrations (3.12 to 100 µg/mL) of TiO_2_, Pd, and gelatin-coated TiO_2_/Pd. The NIH3T3 cell viability decreased as the nanomaterial concentration increased. The gelatin-coated TiO_2_/Pd resulted in lower cell viability than both TiO_2_ and Pd at 100 µg/mL. The cytotoxicity was also determined by AO/EB staining. After AO/EB staining, the fluorescence images of the control group showed uniform green fluorescence of the cell nuclei. By contrast, cell morphology changes, cell-membrane rupture, and cell aggregation occurred for all three sample groups. Hence, gelatin-coated TiO_2_/Pd had a stronger cytotoxic effect than TiO_2_ and Pd (Figure S5).

**Figure 8:**
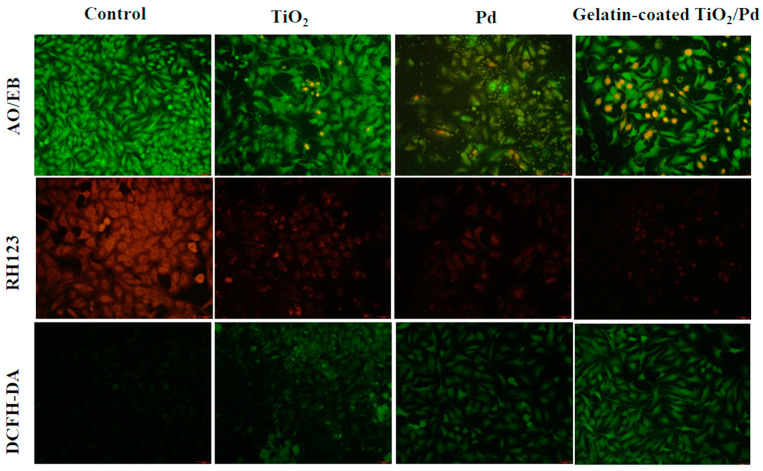
Cytotoxicity of the A549 cells at various concentrations of TiO_2_, Pd, and gelatin-coated TiO_2_/Pd was examined and AO/EB staining by fluorescence microscope (Magnification is 50 µm). Control—The image of the cells in the control medium. Reductions in cell viability were observed by TiO_2_, Pd, and gelatin-coated TiO_2_/Pd.

The cell viability of A549 lung cancer cells was evaluated at different concentrations (3.12 to 100 µg/mL) of TiO_2_, Pd, and gelatin-coated TiO_2_/Pd hybrid nanomaterials. The cell viability was reduced significantly by the gelatin-coated TiO_2_/Pd than TiO_2_ and Pd at 100 µg/mL (Figure 7b). In addition, the cytotoxic effects on A549 cells increased linearly as the nanomaterial concentration increased from 25 µg/mL to 100 µg/mL, indicating a significant reduction in A549 cell viability and demonstrating an anti-cancer effect. The treated A549 cells showed important changes, including cell clumping, nuclear condensation, and shrinkage of the cells. ROS were generated in lung cancer cells after being treated with gelatin-coated TiO_2_/Pd. DCPH-DA fluorescence signal intensity indicates the amount of ROS in gelatin-coated TiO_2_/Pd is greater than in TiO_2_, Pd, and control cells (Figure 8). The level of ROS generation increased, inducing toxicity to the cancer cells and reducing the antioxidant level of the cells. In addition, the high ROS level in cancer cells can cause oxidative stress and DNA damage and disrupt the cellular processes, leading to cell death [36]. In addition, conjugated nanoparticles can have synergistic effects that might involve two roles concurrently to increase toxicity and anticancer activity. This study confirmed that gelatin is involved in conjugating two different kinds of nanomaterials to enhance their antibacterial and anticancer activity.

## 3. Materials and Methods

### 3.1. Materials

Gelatin, titanium dioxide (TiO_2_), and palladium acetate were purchased from Sigma–Aldrich (St. Louis, MI, USA). Ethanol and glycerol were obtained from Samshu Chemicals (Seoul, Republic of Korea). The solutions were prepared using water from a Milli-Q water purification system, and analytical grade chemicals without further purification were used in all experiments.

### 3.2. Material Characterization

XRD (Bruker D2 PHASER, Karlsruhe, Germany) was conducted using Cu Kα radiation (λ = 0.1541 nm). The functional groups on the surface of the samples were confirmed using FT-IR spectroscopy (Jasco FT/IR-6600, Tokyo, Japan) over the wavelength range between 4000 and 400 cm^−1^ using KBr pellets. The sample morphologies and microstructures were characterized by FE-SEM (SU 8010 HR-SEM, Hitachi, Tokyo, Japan)]. HR-TEM (Tecnai G2, FEI, Eindhoven, The Netherlands) furnished with energy dispersive X-ray spectroscopy (EDS) was conducted to observe the morphology and determine the weight percentage of the resulting adsorbent. The sample surface chemical compositions were determined by XPS (Thermo Scientific K-Alpha, Tsukuba, Japan) using an Al X-ray source. The XPS peaks were fitted using CASA XPS software (Version 2.3.25PR1.0), and the binding energies were calibrated to C 1s at 284.6 eV. The zeta potential analyzer (ELC-Z model, Photal Otsuka Electronics, Osaka, Japan) was used to examine the stability of the gelatin-coated TiO_2_/Pd, respectively.

### 3.3. Preparation of the Gelatin-Coated TiO_2_/Pd Nanomaterials

A clear gelatin solution was prepared by dissolving a calculated quantity of gelatin in distilled water and stirring at 60 °C for 2 h. TiO_2_ nanoparticles were then added and stirred continuously for another 60 min. During stirring, a small quantity of glycerol was added to the gelatin/TiO_2_ mixture. Subsequently, palladium acetate was added to the gelatin/TiO_2_ mixture and stirred for approximately 2 h at 60 °C. The solution mixture was then exposed to UV light to reduce palladium acetate to palladium nanoparticles deposited on the gelatin/TiO_2_. The solution was dried for 12 h at 80 °C in a vacuum oven to obtain gelatin-coated TiO_2_/Pd nanomaterials.

### 3.4. Antibacterial Potential

The antibacterial properties of the gelatin-coated TiO_2_/Pd hybrid nanomaterial against *E. coli* and *B. subtilis* were performed using the disc-diffusion method [37,38]. The bacterial strains were grown in Luria–Bertani (LB) broth and kept at 37 °C for incubation. The culture was distributed evenly at 100 µL on an LB agar plate. Sterile discs (6 mm in diameter) of Whatman No. 1 paper were placed on agar plates and impregnated with various concentrations of the gelatin-coated TiO_2_/Pd hybrid (15, 30, and 45 µg/mL). The control paper discs were prepared using sterile water. All treated plates were incubated at 37 °C for 24 h. After inoculation, the plates were incubated at 37 °C for 24 h. Bacterial inhibition was measured by calculating the region surrounding a sterile disc where bacteria could not proliferate. All experiments were done in triplicate.

### 3.5. In Vitro Cell Viability Assay

The cytotoxicity of gelatin-coated TiO_2_/Pd on mouse embryonic fibroblasts (NIH3T3) and lung cancer (A549) cells was determined using in vitro WST (Water Soluble Tetrazolium salt) cell viability assay [39]. Both cells were purchased from the Korean Cell Line Bank. NIH3T3 and A549 were grown in DMEM and RPMI medium supplemented with 10% fetal bovine serum and 1% antibiotics (penicillin and streptomycin), respectively, in a 5% CO_2_ incubator at 37 °C. The specific experimental steps were as follows: The cells (100 μL, 1 × 10^4^) were inoculated into 96-well plates and incubated overnight. The cells in each well of the 96-well plate had to be at least 70–75% confluent before starting the experiment. The prepared gelatin-coated TiO_2_/Pd, TiO_2_, and Pd were added to each well of the plates and incubated for 24 h (final concentrations: 3.12, 6.25, 12.5, 25, 50, and 100 μg/mL). At the end of the incubation, 10 μL of WST was added to each well and incubated for another 1–3 h. WST was cleaved into formazan by cellular mitochondrial dehydrogenases enzyme. The formazan was measured at 450 nm using a 96-well plate reader (SpectraMax^®^ Plus-384 Microplate Reader (Molecular Devices Japan Co., Ltd., Tokyo, Japan)). The cell viability was calculated as a percentage. All experiments were done three times.

Acridine orange–ethidium bromide (AO/EB), rhodamine 123 (Rh123), and DCFH-DA were used for cell staining [40,41]. AO/EB double staining was used to assess the apoptotic behavior of the cells to determine the nuclear variation. Rhodamine 123 (Rh123) was used to observe the mitochondrial membrane potential. DCFH-DA was used to observe reactive oxygen species production. The final concentration of the TiO_2_, Pd, and gelatin-coated TiO_2_/Pd used to treat the cells was 50 μg/mL. Cell staining was imaged and observed under a fluorescence microscope (Olympus, CKX53 culture-microscope, Tokyo, Japan).

## 4. Conclusions

Gelatin-coated TiO_2_/Pd nanomaterials were fabricated to improve their antibacterial and anticancer properties. The hybrid nanomaterials had a spherical shape and a mean size of 17 nm. EDS data revealed that the nanomaterials contained Ti, O, Pd, C, and N. The functional groups and crystalline nature were confirmed by FTIR spectroscopy and XRD, respectively. XPS result confirmed the elemental composition of the gelatin-coated TiO_2_/Pd nanomaterials. Combining different nanomaterials on the nanoscale produced synergistic antibacterial and anticancer activities. The gelatin-coated TiO_2_/Pd nanomaterials significantly affected the growth of *E. coli* (22 mm) and *B. subtilis* (17 mm). The cytotoxicity to A549 lung cancer cells increased linearly as the hybrid nanomaterial concentration increased. Hence, the hybrid nanomaterials might have a synergistic impact that could involve two distinct roles concomitantly to improve the toxicity and anticancer efficacy. The gelatin-coated hybrid nanomaterials could be a useful tool for developing diverse medical applications because they can be tailored specifically to address particular medical challenges in the future.

## Data Availability

The data presented in this study are available on request from the corresponding author.

## Supplementary Materials

The following supporting information can be downloaded at: https://www.mdpi.com/article/10.3390/ijms25105308/s1.
