# Supplementary material for: Gelatin-Coated TiO2/Pd Hybrid: A Potentially Useful Nanomaterial to Enhance Antibacterial and Anticancer Properties
Source: Int J Mol Sci. 2024 May 13;25(10):5308. doi: 10.3390/ijms25105308 (PMC11121089; doi:10.3390/ijms25105308)
Supplement: Supplementary file 1 [file ijms-25-05308-s001.zip › ijms-2957213-supplementary.pdf]

SUPPORTING INFORMATION

Gelatin-Coated TiO_2/Pd Hybrid: A Potentially Useful Nanomaterial to Enhance Antibacterial and Anticancer Properties

Periasamy Anbu [1,*], Muruganantham Rethinasabapathy [2], Anbazhagan Sathiyaseelan [3], Xin Zhang [3], Myeong-Hyeon Wang [3], Sekar Vijayakumar [4] and Yun Suk Huh [2,*]

1 Department of Biological Engineering, Inha University, Incheon 22212, Republic of Korea
2 NanoBio High-Tech Materials Research Center, Department of Biological Sciences and Bioengineering,
Inha University, 100 Inha-ro, Incheon 22212, Republic of Korea; rmaannauniv@gmail.com
3 Department of Medical Biotechnology, College of Biomedical Sciences, Kangwon National University, Chuncheon-si 24341, Republic of Korea; sathiyaseelan.bio@gmail.com (A.S.); zhangxin199708@gmail.com (X.Z.); mhwang@kangwon.ac.kr (M.-H.W.)
4 Marine College, Shandong University, Weihai 264209, China; vijaysekar05@gmail.com
* Correspondence: anbu25@yahoo.com or anbu25@inha.ac.kr (P.A.); yunsuk.huh@inha.ac.kr (Y.S.H.)

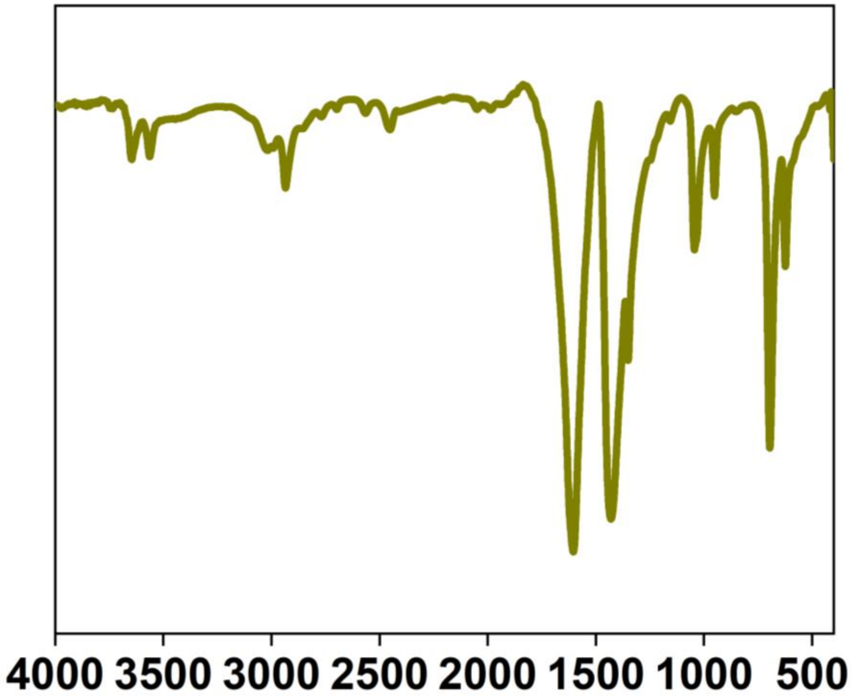

Figure S1. FTIR of palladium acetate.

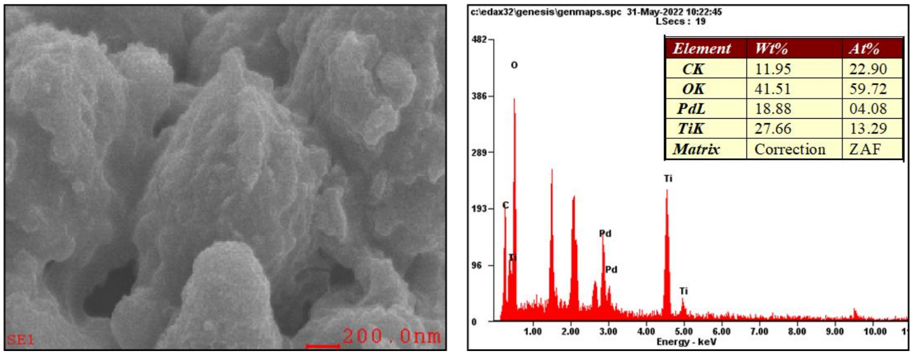

Figure S2. HR-SEM EDX of gelatin-coated TiO_2/Pd hybrid nanomaterials.

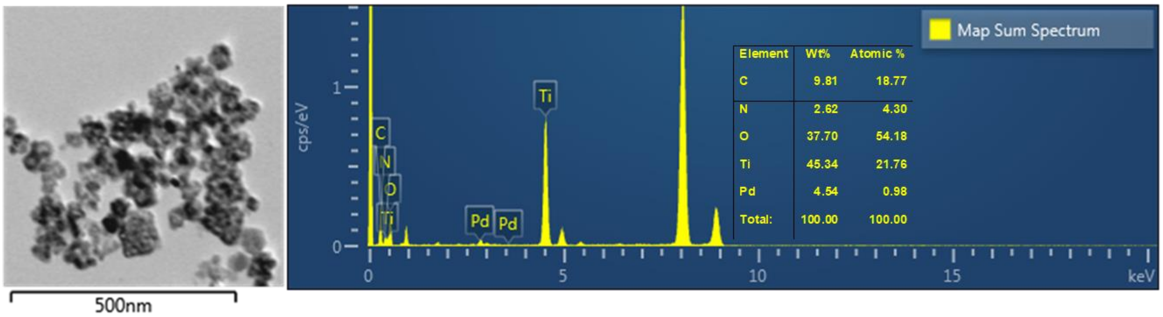

Figure S3. HR-TEM EDX of gelatin-coated TiO_2/Pd hybrid nanomaterials.

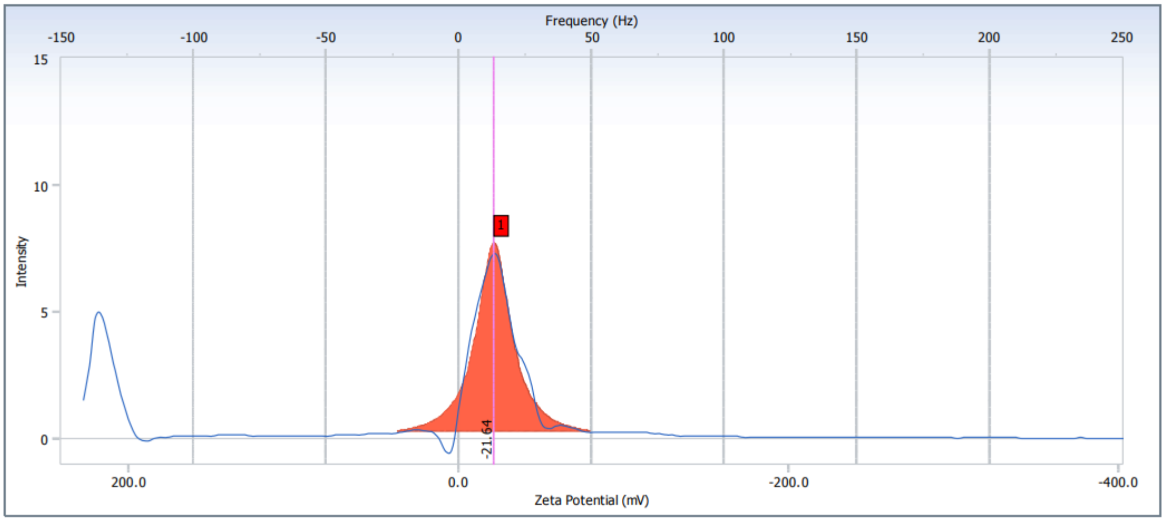

Figure S4. Zeta potential of gelatin-coated TiO_2/Pd hybrid nanomaterials.

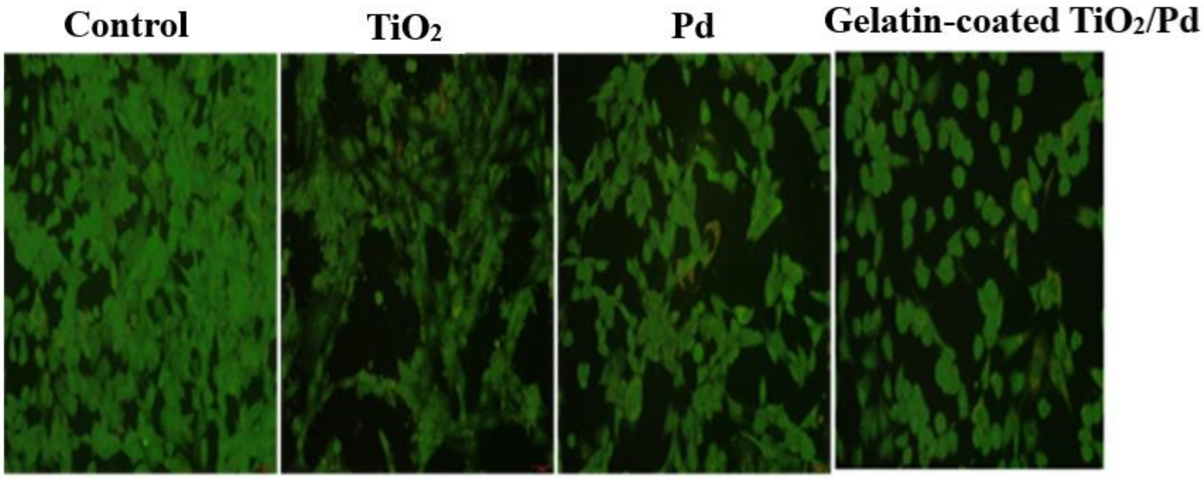

Figure S5. Cytotoxicity of the NIH3T3 cells by TiO_2, Pd and gelatin-coated TiO_2/Pd examined via light microscopy and AO/EB staining. Control—Optical image under a light microscope of cells in control medium. Reductions in cell viability observed by TiO_2, Pd and gelatin-coated TiO_2/Pd.
